# Grain yield genetic gains and changes in physiological related traits for CIMMYT’s High Rainfall Wheat Screening Nursery tested across international environments

**DOI:** 10.1016/j.fcr.2020.107742

**Published:** 2020-04-01

**Authors:** Guillermo S. Gerard, Leonardo A. Crespo-Herrera, José Crossa, Suchismita Mondal, Govindan Velu, Philomin Juliana, Julio Huerta-Espino, Mateo Vargas, Mandeep S. Rhandawa, Sridhar Bhavani, Hans Braun, Ravi P. Singh

**Affiliations:** a Department of Plant Sciences and Crop Development Centre, University of Saskatchewan, 51 Campus Dr., Saskatoon, SK, S7N 5A8, Canada; b Centro Internacional de Mejoramiento de Maíz y Trigo (CIMMYT), Global Wheat Program, Apdo. 0660, Mexico City, Mexico; c INIFAP, Campo Experimental Valle de Mexico, Apdo. Postal 10, Chapingo, Texcoco 56230, Mexico; d Universidad Autónoma Chapingo, Carretera Mexico-Texcoco Km. 38.5, Chapingo, Texcoco 56230, Mexico; e Centro Internacional de Mejoramiento de Maíz y Trigo (CIMMYT), Nairobi, Kenya

**Keywords:** BLUP, best linear unbiased predictor, CGIAR, Consultative Group for International Agricultural Research, DH, days to heading, DM, days to maturity, DHM, days from heading to maturity, FA, factor analytic, GE, genotype × environment interaction, GN, grain number per square meter, GW, grain weight, GY, grain yield, GYLC, grain yield relative to local checks, GYP, grain yield *per se*, HRWYT, high rainfall wheat yield trial, HYL, highest yielding line, IWIN, International Wheat Improvement Network, LC, local check, ME, mega-environment, NASA, National Aeronautics and Space Administration, PH, plant height, POWER, Prediction of Worldwide Energy Resource, High Rainfall Wheat Screening Nursery, Grain yield, Genetic gains, Physiological components, *Triticum aestivum* L.

## Abstract

The effects of climate change together with the projected future demand represents a huge challenge for wheat production systems worldwide. Wheat breeding can contribute to global food security through the creation of genotypes exhibiting stress tolerance and higher yield potential. The objectives of our study were to (*i*) estimate the annual grain yield (GY) genetic gain of High Rainfall Wheat Yield Trials (HRWYT) grown from 2007 (15th HRWYT) to 2016 (24th HRWYT) across international environments, and (*ii*) determine the changes in physiological traits associated with GY genetic improvement. The GY genetic gains were estimated as genetic progress *per se* (GYP) and in terms of local checks (GYLC). In total, 239 international locations were classified into two groups: high- and low-rainfall environments based on climate variables and trial management practices. In the high-rainfall environment, the annual genetic gains for GYP and GYLC were 3.8 and 1.17 % (160 and 65.1 kg ha^−1^ yr^−1^), respectively. In the low-rainfall environment, the genetic gains were 0.93 and 0.73 % (40 and 33.1 kg ha^−1^ yr^−1^), for GYP and GYLC respectively. The GY of the lines included in each nursery showed a significant phenotypic correlation between high- and low-rainfall environments in all the examined years and several of the five best performing lines were common in both environments. The GY progress was mainly associated with increased grain weight (R^2^ = 0.35 *p* < 0.001), days to maturity (R^2^ = 0.20, *p* < 0.001) and grain filling period (R^2^ = 0.06, *p* < 0.05). These results indicate continuous GY genetic progress and yield stability in the HRWYT germplasm developed and distributed by CIMMYT.

## Highlights

• Linear and consistent grain yield genetic gains in the HRWYT has been observed.
• Several best performing lines were common in both high and low rainfall environments.
• The genetic gains were explained by paralleled increases in grain weight, day to maturity and grain filling period.
• These results indicate continuous genetic progress and yield stability in the HRWYT germplasm developed by CIMMYT.

## 1 Introduction

Bread wheat (*Triticum aestivum* L.) is one of the most important crops in the world, providing about 20 % of the daily protein and food calories consumed globally (FAO, 2019). However, based on the projected cereal demand in the coming years wheat production must increase by 60 % (Fischer and Edmeades, 2010). As the wheat grown area may only marginally expand, production goals have to be achieved by improving yield potential, resource use efficiency, and sustainable agronomic practices. Climate change effects such as rising temperatures, frequent seasonal drought and changing patterns of pests and diseases provide additional challenges for ensuring yield stability across diverse environments and meet the future demand for wheat products. In this context, wheat breeding can make a significant contribution to global food security through the creation of high-yielding and stress tolerant genotypes, which adequately respond to expected future climatic conditions (Crespo-Herrera et al., 2018).

The CIMMYT bread wheat breeding program uses a shuttle breeding strategy together with the possibility to simulate different growing conditions at the main yield-testing site in Ciudad Obregon, Mexico. This strategy allows the development of new high-yielding and stress tolerant lines, targeted to more than 60 million hectares of spring wheat around the world. To improve the selection efficiency and address the needs of diverse wheat growing areas, CIMMYT uses multi-location testing and the concept of mega-environments (ME) to target germplasm development. A ME, seen also as a target population environment, is defined as a broad, not necessarily contiguous area, occurring in more than one country and frequently transcontinental, defined by climate, soil, similar biotic and abiotic stresses, cropping system requirements, etc. (Rajaram et al., 1993; Hodson and White, 2007). There are twelve MEs defined for wheat production, of which ME1 to ME6 correspond to spring wheat. The ME2 comprises the so-called high rainfall areas, where the precipitation is ≥150 (ME2a) and ≥250 (ME2b) mm and the average minimum temperature is between 3 and 16 °C during the wettest quarter. The major constraints in ME2 are foliar diseases such as stripe rust (*Puccinia striiformis* f. sp. *tritici*), Septoria tritici blotch (*Zymoseptoria tritici*) and tan spot (*Pyrenophora tritici-repentis*) (Hodson and White, 2007). The ME2 covers 5 million hectares, including the central highlands of eastern and Central Africa, North Africa, West Asia, the Southern Cone and Andean highlands of South America, and the highlands of Central Mexico (Tadesse et al., 2010).

In response to the needs of farmers in high rainfall areas, CIMMYT annually distributes the High Rainfall Wheat Screening Nursery (HRWYT) to more than 100 collaborators worldwide within the International Wheat Improvement Network (IWIN). The germplasm carries the required characteristics for variety release or use as progenitors by local breeding programs. Although HRWYT targets high rainfall areas, often they are grown in regions classified within other MEs as per collaborators request, since the yield stability of CIMMYT germplasm often allows them to find lines that also perform well in others MEs. Cooperators evaluate this elite germplasm at several locations across the world and return the phenotypic data to CIMMYT. Information from such trials is strategic for CIMMYT as it helps breeders to make better breeding decisions by selecting superior parents; and it allows the estimation of grain yield (GY) genetic gains achieved over a certain period, as a measure of breeding efficiency.

This type of evaluations are imperative due to the significant impact of CIMMYT wheat germplasm on world wheat production, given that 70 % of cultivars grown in target countries are either direct CGIAR releases or used as parents (Lantican et al., 2016), and since the world scenario requires the increase of the yield potential as the main priority. In a previous study, Tadesse et al. (2010) looked at the genetic progress for GY from the first through the 12th HRWYT (1992–2004). They identified several well-adapted CIMMYT lines based on its success rate (percentage of sites where the line was yielding higher than the local checks). However, as proposed by Singh et al. (2007), a regular assessment and upgrade of the yield progress are necessary to evaluate the performance of breeding programs on a global scale and incorporate new breeding strategies adapted to the new scenarios.

In addition to assessing the GY genetic gains, studying the genetic base and the changes in yield-related traits is valuable to recognize which of them are associated with GY, identify yield-limiting factors and specially to plan future effective approaches to increase the GY genetic gains in breeding programs. Historical studies have shown that traits such as grains per square meter, biomass, harvest index, and reduced plant height are positively associated with GY progress (Slafer et al., 1990; Shearman et al., 2005; Xiao et al., 2012). However, more recent studies carried out particularly with CIMMYT material suggest that GY genetic gains are mainly associated with flowering time, grain size and grain weight (Lopes et al., 2012; Aisawi et al., 2015).

In general, complex traits such as GY and its components show Genotype-by-Environment interaction (GE), which may be expressed as heterogeneity in genetic variance amongst environments (scale changes) and/or in the ranking of individuals (crossover interaction) (Burgueño et al., 2008). Genotype by environment interactions can reduce trait heritability and the ability to predict statistically superior genotypes under contrasting environments. Several structures for modeling the GE can be used, but currently, the factor analytic (FA) models, which separate genetic effects into common and specific components, seems to be the superior form for increasing the accuracy of genotypic selection in plant breeding multi-environment trials (Crossa et al., 2006; Burgueño et al., 2007). In the present study, 10 years of historical performance data for international High Rainfall Wheat Yield Trails (HRWYTs) were analyzed by fitting the FA model structure. The objectives of our study were to (*i*) estimate the annual GY genetic gain for High Rainfall Wheat Yield Trial grown from 2007 (15th HRWYT) to 2016 (24th HRWYT) across international environments, and (*ii*) determine the changes produced by GY genetic improvement on associated physiological traits.

## 2 Materials and methods

### 2.1 Plant material and crop environments

Each HRWYT consisted of 30–50 entries, one of which is a local check (LC) included in the trial by each collaborator (often the best commercial variety) and the remaining entries are new CIMMYT’s elite wheat lines and checks. The elite lines are selected after 2 years of testing under optimal irrigated conditions and an additional 1–2 years under drought and heat-stressed conditions at CIMMYT’s experimental station in Ciudad Obregon, Mexico (27° 37’ N, 109° 93’ W). Germplasm selected for assembling HRWYTs each year are multiplied at Mexicali, Baja California Norte, a Karnal bunt (*Tilletia indica*) free site in Northwestern Mexico (32° 39′ N, 115° 28′ O). Then, the yield trial nursery is distributed globally as requested by cooperators. The trial design is an α-lattice with two replicates. New lines are distributed each year, hence the composition of entries is different from year to year.

The GY analyzed data set in the present study belongs to the 15th through 24th HRWYTs distributed and grown from 2007 to 2016 at 360 locations in 53 countries. Based on climate variables used to classify the ME by Hodson and White (2007) (Average minimal temperature and total precipitation for the wettest quarter) and together with the trial management information provided by cooperators (sowing date, altitude, etc.) the locations in each year were assigned to two groups: high- and low-rainfall environments (Table 1). The average minimal temperature and total precipitation for each location were obtained from the NASA Langley Research Center (LaRC) POWER Project funded through the NASA Earth Science/Applied Science Program.

**Table 1:** Climatic variables and criteria used to assign the analyzed sites in both high and low rainfall environments.

Mega-Environment	Precipitation (mm)*		Mean min Temp (°C)*		Altitude (m)	
	range	average	range	average	range	average
High Rainfall Environment	>150	282	−5.74/19.34	9.34	3/3200	781
Low Rainfall Environment	<150	53	−0.51/24.36	9.87	3/2090	329

* Precipitation and mean minimum temperature in the coolest quarter (three consecutive coolest months of the year).

The physiological yield related traits were taken by cooperators according to the Instructions for the Management and Reporting of Results for CIMMYT International Wheat Nurseries (See Supplementary Material S1). Briefely, days to heading (DH) was considered when 50 % of the plants in a plot had spikes completely emerged from the flag leaf. Similarly, days to maturity (DM) was considered when 50 % of the spikes in a plot had lost all green coloration. Days from heading to maturity (DHM) were calculated by subtracting the time to maturity from the time to heading. Plant height was determined by measuring the plant height in centimeter, excluding awns, on two measurements per plot. Plots are harvested soon after physiological maturity, GY was recorded in grams per square meters and then converted into tons per hectare. Grain weight was determined using two samples of 200 grains were randomly taken from each plot and weighed on a precise balance. Finally, GN was calculated as the quotient between GY and GW.

The physiological yield related traits were analyzed, but only in high-rainfall environment from 2010 (18th) to 2016 (24th), because data was not available in the previous years.

Regarding the management practices carried out and data returned by cooperators, almost all trials were fertilized (99 %), while the 96 % reported having used fungicides. In line with the reported fungicide treatments, only 8 % of the trials showed severe disease development and 29, 20, 27, and 16 % displayed moderate, slight, traces and no foliar disease development, respectively (Supplementary Fig. S1). In all reported data trials, weeds were well controlled, since 53 % did not report weed problems, and 14, 16, 15 and only 1 % reported traces, slight, moderate, and severe weed problems, respectively. Of all the sites, 30 % were reported to be irrigated and 2 % had severe lodging, while 6, 13, 22, and 57 % showed moderate, slight, traces and no lodging problems, respectively. The average experimental plot was 4.6 m^2^.

### 2.2 Statistical analysis

#### 2.2.1 Individual site linear mixed model

The linear mixed models used in the statistical analyses are in line with those used by Crespo-Herrera et al. (2017, 2018) and were computed using ASREML-R (Butler et al., 2009) in R version 2.15.3 software (R Development Core Team, 2013). The first step of the analysis consisted of analyzing all the sites individually to estimate heritability (H^2^) using the following statistical model:

(1) $$Y_{ijk}=\;\mu +\;R_{j}+\;B_{k}\left(R_{j}\right)+\;G_{i}+\;\varepsilon_{ijk}$$

where μ is the general mean, *R_j_* is the fixed effects of the replicates (*j* = 1, 2), *G_i_* is the fixed effects of the wheat lines (*i* = 1, …, 50), and *B_k_* represents the random effects of the incomplete -blocks (*k* = 1, …, 5), assumed to be independently and identically normal distributed (iid) with mean zero and variance σ^2^*_sb(r)_*. The term ε*_ijk_* is a random residual assumed to be iid with mean zero and variance σ^2^_ε_. The heritability was estimated using the variance components of each site and those sites with H^2^ <0.05 were excluded from further analysis. Then, a k-means clustering analysis using the POWER weather data of each remained trial was used to define the designation of the sites in each of two high- and low-rainfall environments. Once the sites were grouped, data from each HRWYT were analyzed in a combined analysis across locations and by group.

#### 2.2.2 Multi-site linear mixed model

The linear mixed model used in the multi-site trial analysis can be written as:

(2) $$Y={1\mu +X}_{s}s+Z_{r}r+\;{Z_{b}b+Z}_{g}g+Z_{ge}ge+e$$

where **1** is a column vector of ones, μ is the overall mean, ***X_s_*** is the incidence matrix for the fixed effects of sites, and ***Z_r_, Z_b_, Z_g_,*** and ***Z_ge_*** are the design matrices for the random effects of replicates within sites, incomplete blocks within replicates and sites, genotypes, and GE, respectively. Vector ***s*** denotes the fixed effect of sites; while vectors ***r*, *b*, *g*, *ge***, and ***e*** contain random effects of replicates within sites, incomplete blocks within replicates and sites, lines, GE, and residuals, respectively, and they are assumed to be iid random variables, normally distributed with zero mean vectors and variance-covariance matrices ***R, B, G, GE***, and ***E,*** respectively.

The variance-covariance matrices ***R, B, G,*** and ***E*** are simple variance-covariance component with ***R*** = $\sigma_{r}^{2}$ ***I_r_***, ***B*** = $\sigma_{b}^{2}$ ***I_b_***, ***G*** = $\sigma_{g}^{2}$ ***I_g_*** and ***E*** = $\sigma_{e}^{2}$ ***I_rsg_***, where $\sigma_{r}^{2}$, $\sigma_{b}^{2}$, $\sigma_{g}^{2}$, $\sigma_{e}^{2}$ are variance components of the replicates within site, incomplete blocks within replicates and sites, line, and residual, respectively multiplied by their corresponding identity matrices ***I***. The variance-covariance matrix ***GE*** is the variance of the line effects in individual environments and can be represented as the Kronecker product between the variance-covariance matrix for the dimension of the sites ($\sum_{s}$) and the variance-covariance matrix for the dimension of the genotypes (***I_g_***): ***GE*** = $\sum_{s}$ **⊗ I_g_**. In this study, we modeled the environmental component, $\sum_{s}$, using the factor analytic (FA) model, and assumed that in the identity matrix, ***I_g_***, the genotypes are iid (Crossa et al., 2006). Thus, best linear unbiased predictors (BLUP) were obtained for each line in each environment for each year of testing. Best linear unbiased predictors were estimated independently for each of the physiological related traits, *e.g.*, DHM was calculated from the raw data and then analyzed with the mixed models framework. This type of analysis for combining data across sites for measuring genetic gains across years was used by Crespo-Herrera et al. (2017, 2018).

### 2.3 Measuring genetic gains using the line means across sites based on the factor analytic model

The GY genetic gains were determined as genetic progress *per se* (GYP), in which the GY BLUPs of the five highest-yielding lines (HYL), estimated across all locations within each year, were regressed over the years of HRWYT evaluation. Here, the percentage of the genetic gain was calculated over average yield across the 10 years analyzed. In addition, we also determined the genetic progress of the CIMMYT’s line in terms of LCs as the regression slope of the five HYL GY BLUP minus the mean yield of LCs across all locations divided by the grain yield of the local check of HRWYT evaluation (GYLC),

$$GYLC=\;\frac{BLUP-LCGY}{LCGY}*100$$

where *BLUP* represents the average GY BLUP value of CIMMYT’s five HYL, estimated across locations within each year, and *LCGY* represents the predicted GY BLUP mean of the LCs.

On the other hand, the association between GY and physiological related traits was tested and illustrated using biplot, whereas multiple regression analyses were performed to determine the effects and relative contribution of each trait to GY in the high-rainfall environment.

## 3 Results

### 3.1 High and low rainfall wheat yield trial distribution

**Fig. 1:**
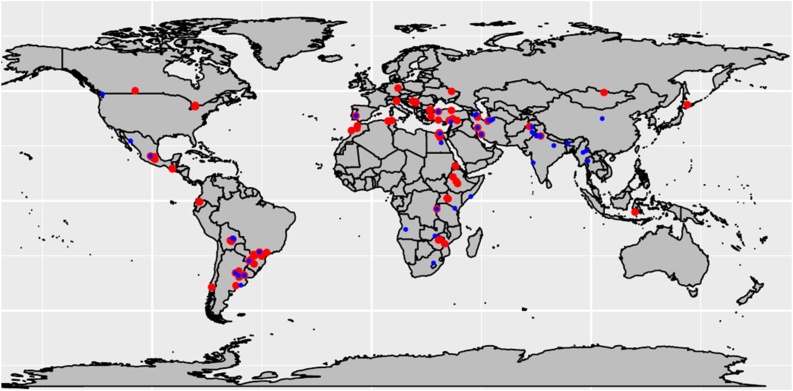
Locations contributing High Rainfall Wheat Yield Trials (HRWYT) data for analysis from 2007 (15th HRWYT) to 2016 (24th HRWYT). Red and Blue points indicate high- and low-rainfall environments, respectively. Some locations show both points (red and blue) because they have been classified in both high- and low-rainfall environments depending on the year.

Of the 360 sites, 239 (66 %) with complete data and heritability higher than 0.05 were used for further analysis. These trials were distributed across Asia (28.5 %), South America (22.2 %), Africa (19.2 %), North America (18.4 %) and Europe (11.7 %). Based on the POWER weather data and trial management information provided by cooperators, the k-means clustering analysis grouped 138 sites (57.7 %) to the high-rainfall (well-watered) environment, and 101 (42.3 %) belonged to the low-rainfall environment (Table 2; Fig. 1).

**Table 2:** Number of locations in the two environments where the 15^th^ to 24^th^ High Rainfall Wheat Yield Trials were analyzed.

Environments	HRWYT										
	15th	16th	17th	18th	19th	20th	21th	22th	23th	24th	Total
High-Rainfall	9	10	10	17	11	18	13	15	20	15	138
Low-Rainfall	7	8	11	4	10	7	11	13	11	19	101
Total	16	18	21	21	21	25	24	28	31	34	239

### 3.2 Grain yield performance and genetic gains

**Fig. 2:**
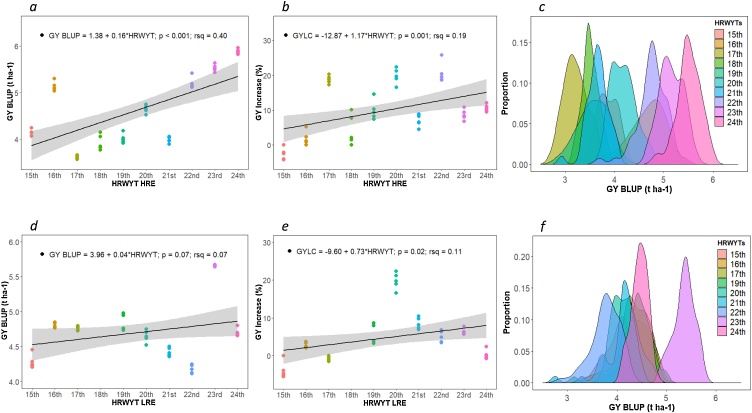
Annual grain yield (GY) genetic gain of CIMMYT’s High Rainfall Screening Nursery grown from 2007 (15th HRWYT) to 2016 (24th HRWYT) across international environments. a and d) Grain yield genetic progress *per se* (t ha-1) for the five highest yielding lines during the analyzed period; b and e) Percentage of GY genetic progress relative to local checks (GY_LC) for the five highest yielding lines during the analyzed period and c, f) GY density distribution of all CIMMYT’s line included in each nursery, tested in high-rainfall environment (HRE) and low-rainfall environment (LRE), respectively.

The mean GY of the five HYL across all HRWYT in high-rainfall environment was 4.60 t ha^−1^ and ranged from 3.57 (17th) to 5.97 t ha^−1^ (24th) (Fig. 2a), whereas in low-rainfall environment the mean GY was 4.30 t ha^−1^, ranging from 4.11 (22nd) to 5.75 t ha^–1^ (23rd) (Fig. 2d). Meanwhile, the GY of LCs ranged from 3.04 (17th) to 5.32 t ha^−1^ (24th) and from 3.88 (20th) to 5.34 t ha^−1^ (23rd) in high- and low-rainfall environments, respectively. In addition, the GY of the lines included in each nursery, showed a significant (*p* < 0.01) phenotypic correlation between high- and low-rainfall environments ranging from *r* = 0.41 (22nd) to *r* = 0.83 (15th). The performance of the HYL expressed as a GY BLUP, percentage of GY increase relative to LCs, and GY rank across high- and low-rainfall environments are given in Supplementary Table 1. In general, the outstanding lines performed well in both environments, in fact, 26 % of the best performing lines in the high-rainfall environment were also amongst the best performing lines in the low-rainfall environment (Supplementary Table 1).

Significant genetic progress for GYP and GYLC was observed in both environments by modeling the GE interaction with the FA structure (Fig. 2).

In the high-rainfall environment, the annual rate of GYP increase was 3.8 %, which represents 160 kg ha^−1^ yr^−1^ (Fig. 2a). When the GY of the HYL was compared with LCs (Fig. 2b), the analysis indicated 1.17 % of genetic progress, which represents 65.1 kg ha^−1^ yr^−1^. In addition, as shown in Fig. 2c the density plot including all HRWYT lines displays substantial GY increases over time. For both, GYP and GYLC, the slope of the regression was highly significant (*p* ≤ 0.001), with R^2^ of 0.40 and 0.19, respectively.

In the low-rainfall sites, the mean GYP of the HYL displayed 0.93 % genetic progress per year, which represents 40 kg ha^−1^ yr^−1^ (Fig. 2d). Even though the trend for GYP was positive, this was not significant (p = 0.07). The trend in the mean grain yield of the HYL relative to LCs exhibited 0.73 % (33.1 kg ha^−1^ yr^−1^) of yield gain (Fig. 2e). In this environment, the density plots also showed a general GY increase over the years (Fig. 2f), although the determination coefficients were lower.

### 3.3 Physiological traits performance in high rainfall environment

**Fig. 3:**
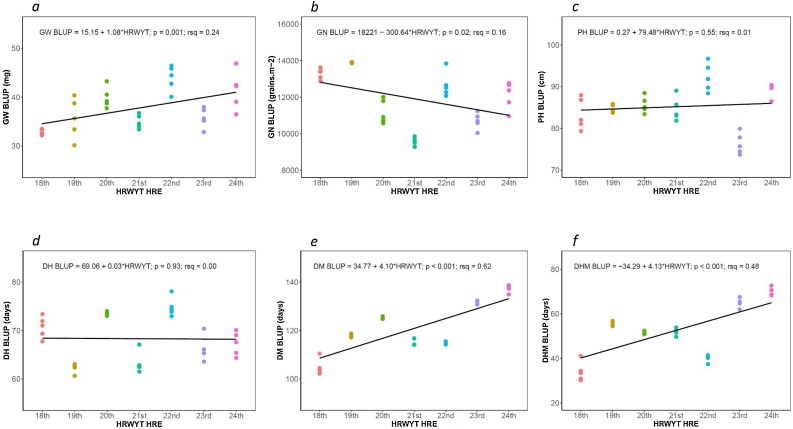
Changes in physiological related traits for the five highest yielding lines of CIMMYT’s High Rainfall Wheat Screening Nursery grown in high-rainfall international environments from 2010 (18th) to 2016 (24th). (a) GW, grain weight; (b) GN, grain number per m^−2^; (c) PH, plant height; (d) DH, days to heading; (e) DM, days to maturity; and (f) DHM, days between heading and maturity.

Overall, GW ranged from 30.1 (19th) to 46.9 (24th) mg, showing a strong and positive linear relationship when it was plotted against years (Fig. 3a). Thus, the GW means of the five HYL gave an annual increase rate of 1.08 mg yr^−1^ (*p* = 0.001).

The GN ranged from 9279 21 st to 13922 19th grains m^−2^ and presented a negative linear relationship (−300*HRWYT, *p* = 0.020).

Plant height ranged from 73.7 (23rd) to 96.7 (22nd) cm, while DH showed phenotypic values from 60.7 (19th) to 78.1 (22nd) days (Fig. 3c and d). Although the trends were opposite for these two traits, no significant (*p* = 0.55 and *p* = 0.93, respectively) breeding effect was detected when they were regressed over HRWYT years.

Similar trends were observed for DM and DHM, which ranged from 102.0 (18th) to 138.7 (24th) and from 30.2 (18th) to 72.6 (24th) days, respectively. Both trends were significant (*p* < 0.001) and the respective annual rates of progress were 4.10 and 4.13 days yr^−1^, respectively (Fig. 3e and f).

**Fig. 4:**
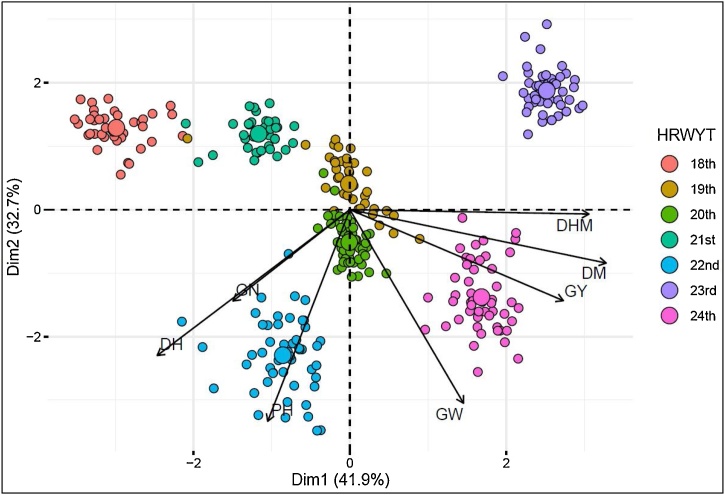
Biplot of grain yield (GY) association with grain weight (GW), grain number (GN), plant height (PH), days to heading (DH), days to maturity (DM), and days from heading to maturity (DHM) in high-rainfall environment.

The association between GY and yield related traits measured in this study are shown as a biplot (Fig. 4). This analysis confirms that the observed positive correlations of GW, DM, and DHM through the years (Fig. 3a, e, and f), were associated with the substantial GY increases over time. Thus, the GY was positively associated with GW, DM, and DHM, while GN, PH, and DH did not show a strong association with the mentioned trait. The first two dimensions of the biplot explained a total of 75.4 % of the variation. These results are in line with the multiple regression analysis, in which only GW, DM, and DHM contributed significantly to explain the GY. The total R^2^ explained by the multiple regression analysis between GY and related traits was 0.68 (*p* < 0.001). The individual trait contributions ranged from 0.9–38.9%, being GW and DM the most significant (Table 3).

**Table 3:** Regression analyses of grain yield (GY) with grain weight (GW), grain number per square meter (GN), days to maturity (DM), days from heading to maturity (DHM), days to heading (DH) and plant height (PH).

High-rainfall environment	R^2^	partial R^2^	Contribution (%)
***GY*** = 0.84 + 0.09 ***GW*** - 0.00 ***GN*** + 0.04 ***DM*** + 0.05 ***DHM*** - 0.02 ***DH*** + 0.00 ***PH***	0.68***		100
***GW***	0.35***	0.35***	38.9
***DM***	0.55***	0.20***	21.3
***DHM***	0.61*	0.06*	6.7
***DH***	0.65	0.04	4.9
***GN***	0.67	0.02	1.9
***PH***	0.68	0.01	0.9

Significance levels: *** p < 0.001, ** p < 0.01, * p < 0.05.

## 4 Discussion

Several methodologies can be used to estimate GY genetic gains. In general, no single method or analysis pipeline will be appropriate for all situations, and their choice depends on various factors such as objectives, resources, the structure of the existing breeding program data, etc. (Rutkoski, 2018). In the present study, we estimated the rate of breeding progress both as genetic progress *per se* and in terms of LCs. As Singh et al. (2007) mentioned, the estimations in terms of local checks are an interesting way to measure genetic gains because the cooperators usually use the best locally adapted commercial variety at the individual site and they continue to change over time. Thus, raising the standard for selecting high-performing lines, although they can introduce noise in the multi-environmental analysis, as LCs can be different in different sites.

In addition, in order to increase the ability to statistically identified high-performing genotypes under contrasting environments and increase precision, we modeled the GE by using the FA structure (Burgueño et al., 2011). Factor analytic models separate genetic effects into common and specific components, increase the accuracy of genotypic selection in plant breeding multi-environment trials, producing lower standard error of the BLUPs, and substantially reducing computational requirements of mixed model analyses compared to standard multivariate models (Crossa et al., 2006; Burgueño et al., 2007).

Our study shows reliable genetic progress for GY over time when the data is expressed as genetic progress *per se* or as a percentage of LCs. The trends observed in the high-rainfall environment were very consistent, exhibiting regression slopes highly significant (*p* ≤ 0.001) and genetic rates of 3.8 % and 1.17 % for GYP and LCs, respectively. Similar genetic progress *per se* has been observed in some countries located in high-rainfall environments. For example, in Ethiopia, the national production showed an annual yield gain of 6.4 % considering the same period analyzed in this study (FAO, 2019). The genetic gains relative to LCs are in line with earlier studies carried out in high rainfall areas, which have reported rate of genetic GY increase across sites between 0.8–1.1% yr^−1^ (Waddington et al., 1986; Sayre et al., 1997).

Although HRWYT germplasm targets high rainfall environments, we also found genetic progress in the low-rainfall environment for the GYLC. In this environment, the genetic progress was lower, reaching rates of 0.93 % and 0.73 % when the genetic gains were expressed as GYP and as a percentage of LCs, respectively. However, the regression slope was only significant for the GYLC (*p* = 0.02), but not as significant as in the high-rainfall environment. This is attributed to the fact the HRWYT germplasm is particularly selected to target sites where precipitation is not a constraint for wheat production. Regarding rainfed areas, the genetic gains reported have been more variables ranging from 0.3 to more than 3 % yr^−1^ (Jain and Byerlee, 1999; Byerlee and Moya, 1993).

Our results are also in agreement with extensive analyses conducted on international yield trials, which show continuous genetic progress in CIMMYT spring wheat in recent decades. Manès et al. (2012) using as common check cultivars Dharwar Dry and Cham 6 reported grain yield increases of 0.5–1.1% and 1.2–1.7% for Semi-Arid Wheat Yield Trials in low- and high-yield environments, respectively. In addition, Sharma et al. (2012) testing the Elite Spring Wheat Yield Trials found a rate of yield increase of 0.55 to 0.62 associated with ME1 and ME2, respectively. Finally, Crespo-Herrera et al. (2017, 2018) using also the FA for assessing the GE interaction, have recently reported genetic gains in the order of 0.41–1% relative to the local checks for Elite Spring Wheat Yield Trials. They also reported gains of 1.41–1.8% relative to the common checks when they tested the performance of Semi-Arid Wheat Yield Trial across the low- and medium-yield environments, respectively. All these results confirm that the CIMMYT wheat breeding program develops new materials with yield stability and higher yield potential.

In some of the analyzed years and contrary to expectations, the GY of HYL was higher in LRE than in HRE. A possible explanation of these results is that in some LRE sites (classified based on the amount of rainfall), artificial irrigation was used, however, many cooperators did not report the amount of water applied.

The GY of the lines included in each nursery showed a significant phenotypic correlation between high- and low-rainfall environments across all the years with R^2^ ranging from 0.41 to 0.83. In this sense, the 26 % of the best performing lines in the high-rainfall environment were also amongst the best in the low-rainfall environment (Supplementary Table 1), which is an indicator that outstanding lines have yield stability across multiple locations. Yield stability is one of the features of CIMMYT germplasm achieved through the strategy of shuttle breeding, together with the possibility to simulate various growing conditions at the main yield-testing site in Ciudad Obregon, Sonora, Mexico (Crespo-Herrera et al., 2018).

Interestingly, the parentage base of the best performing lines common in both environments included CIMMYT lines such as Vorobey, Babax, Kauz, Weebill, and Pastor, as well as the European winter wheat lines Premio, Mercato and Altigo. Those CIMMYT genotypes have been widely reported to maintain stable and superior performance over a range of low, intermediate, and high yield environments, irrespective of irrigation or drought stress (Singh et al., 2007). While the European lines contribute to increasing the resource use efficiency together with stable and high yields under different environmental conditions (Cormier, 2015; Pennacchi et al., 2019). This result supports previous conclusions that CIMMYT breeders successfully broadened the genetic diversity of the elite germplasm through the incorporation of materials from all over the world as well as via primary synthetics into the breeding program (Warburton et al., 2006; Dreisigacker et al., 2008).

Grain yield is a complex trait and a function of several related traits. In the present study, the regression analysis showed a significant contribution to GY from GW, DM, and DHM in the high-rainfall environment. The average annual rate of GW, DM and DHM were 1.08 mg yr^−1^, 4.10 and 4.13 days yr^−1^, and they explained 38.9 %, 21.3 % and 6.7 % of the GY phenotypic variation, respectively (Table 3). Grain number per unit area decreased by an average of 300 grains m^-2^ yr^-1^ and was negatively associated with GY increases, whereas no substantial changes over time were observed for PH and DH, both showing a slightly positive but not significant trend. The absence of variation in PH and DH can be explained due to the shuttle breeding strategy, which results in the selection of photo insensitive wheat genotypes. This intensive selection conducted during the 20th century has generated that important alleles controlling highly heritable traits such as heading and height (*Ppd*-D1a, *Vrn*-B1a, *Vrn*-D1, *Rht*-B1b) are almost fixed in the elite germplasm (Sehgal et al., 2015).

The increase in DHM and DM may be the result of the reduction in the foliar disease levels observed over the years (Supplementary Fig. S1) due to the improvement in disease resistance along with genetic variation in the length of DM and DHM in the analyzed material. Both, the importance of genetic resistance in high rainfall zones, where the foliar diseases pressure is higher than in dryer environments, as well as a significant genotypic variation for DM and DHM in spring wheat have been reported (Zhang et al., 2006; Monpara, 2011; Ullah et al., 2014).

There is evidence that GY could be substantially improved in high-rainfall regions if early-sown, long-season wheat lines are grown to take advantage of the longer growing season compared with the traditional varieties grown in the low and medium-rainfall region (Zhang et al., 2006; Sylvester-Bradley and Riffkin, 2008).

Despite the positive association observed between DHM and DM with grain yield, they did not show a strong association with GW, explaining together only 15 % of its phenotypic variation (*p* = 0.03). As reported by Zhang et al. (2010), these results would indicate that GW in high-rainfall areas would not be limited by source and that the increases observed in GW would be associated with the increase in potential grain size. These results are in line with those reported by Aisawi et al. (2015), who showed that potential grain weight in CIMMYT germplasm has increased and contributed to the genetic gains in final grain weight since GW in the degrained spikes (without sink limitation) increased linearly over the years. Fischer (2007) and Reynolds et al. (2007) manipulating the sink-source balance also suggested that the grain yield in wheat is sink-limited and that increasing the sink capacity would increase the yield potential.

Historical studies have shown that traits as GN, biomass, harvest index (HI) and reduced pH were positively associated with yield progress (Slafer et al., 1990; Shearman et al., 2005; Xiao et al., 2012). The introduction of semi-dwarf cultivars during the 1960s was associated with a higher HI, increasing partition to the spikes during the pre-anthesis phase and improving the grains per square meter. However, more recent studies carried out on CIMMYT material, and in agreement with the results presented here, suggest that during the last period, GY genetic gains are mainly associated with flowering time and GW (Lopes et al., 2012; Aisawi et al., 2015). They found that increases in biomass have contributed to increasing GY through heavier grains rather than more grains per square meter. These increases in GW in CIMMYT material over the last years, at least in part, can be the result of visual selection for grain features such as grain size and plumpness carried out in the F_5_ or F_6_ generation. In the present study, contrary to that reported by Aisawi et al. (2015), the increase in GW was not associated with an increase in PH, which means that future gains in yield potential could be achieved using the current breeding strategy without modifying this trait.

## 5 Conclusions

In agreement with previous reports, our results show a linear and consistent genetic progress in the wheat breeding lines from the HRWYT in both analyzed environments over the last 10 years. The best performing lines were associated with pedigrees that have been widely reported as stable and high-yielding lines across different environments. The present study also revealed that the genetic yield potential improvement of HRWYTs over time was associated with paralleled increases in GW, DM, and DHM as these traits were strongly correlated with GY genetic gains.

## Author statement

**Guillermo S. Gerard**: Writing - Original Draft, Formal Analysis and Methodology**. Leonardo A. Crespo-Herrera**: Conceptualization, Methodology, Resources and Investigation. **José Crossa**: Formal Analysis and Methodology. **Suchismita Mondal**: Investigation and Resources. **Govindan Velu**: Investigation and Resources. **Philomin Juliana**: Investigation and Resources. **Julio Huerta‑Espino**: Investigation and Resources. **Mateo Vargas**: Formal Analysis. **Mandeep S. Rhandawa**: Investigation. **Sridhar Bhavani**: Investigation. **Hans Braun**: Project administration, Funding acquisition; **Ravi P. Singh**: Project administration, Funding acquisition, Investigation, Resources and Supervision. All authors: Writing - Review & Editing

## Declaration of Competing Interest

The authors declare that there is no conflict of interest.
